# Self-Referential Thinking, Suicide, and Function of the Cortical Midline Structures and Striatum in Mood Disorders: Possible Implications for Treatment Studies of Mindfulness-Based Interventions for Bipolar Depression

**DOI:** 10.1155/2012/246725

**Published:** 2011-09-25

**Authors:** William R. Marchand

**Affiliations:** ^1^Research Service, George E. Wahlen Department of Veterans Affairs Medical Center, Salt Lake City, UT 84148, USA; ^2^Department of Psychiatry, The University of Utah, Salt Lake City, UT 84112, USA

## Abstract

Bipolar depression is often refractory to treatment and is frequently associated with anxiety symptoms and elevated suicide risk. There is a great need for adjunctive psychotherapeutic interventions. Treatments with effectiveness for depressive and anxiety symptoms as well as suicide-related thoughts and behaviors would be particularly beneficial. Mindfulness-based interventions hold promise, and studies of these approaches for bipolar disorder are warranted. The aim of this paper is to provide a conceptual background for such studies by reviewing key findings from diverse lines of investigation. Results of that review indicate that cortical midline structures (CMS) appear to link abnormal self-referential thinking to emotional dysregulation in mood disorders. Furthermore, CMS and striatal dysfunction may play a role in the neuropathology underlying suicide-related thoughts and behaviors. Thus, combining studies of mindfulness interventions targeting abnormal self-referential thinking with functional imaging of CMS and striatal function may help delineate the neurobiological mechanisms of action of these treatments.

## 1. Introduction

The neurobiology of bipolar spectrum disorders is incompletely characterized [1]. Further, current medication treatments are only partially effective [2], and 73% of patients receiving pharmacotherapy relapse within 5 years [3]. Thus, there is a compelling need for effective adjunctive psychotherapy interventions [4, 5]. Cognitive-behavioral therapy is an effective intervention for depression [4, 6–9] but seems to be of less benefit for bipolar spectrum illness [10]. Bipolar disorder is highly comorbid with anxiety [11, 12], and therefore interventions that also target anxiety might be of particular benefit [13]. Mindfulness-based psychotherapies are being increasingly used to target both depressive and anxiety symptoms in a variety of clinical populations [14–16]. However, few studies [17–19] have investigated these interventions for bipolar spectrum disorders. Furthermore, an integrated conceptual framework for such studies has not been published.

Mindfulness interventions target aberrant self-referential thinking [20]. Increasing evidence suggests that alterations in self-referential thinking may be associated with a number of mood and anxiety spectrum disorders. For example, investigations indicate an association between aberrant self-concept and/or self-schemas and unipolar depression [21–25], generalized anxiety disorder [26], obsessive-compulsive disorder [27, 28], PTSD [29–31], social phobia [32, 33], and panic disorder [34]. There is evidence that anomalous self-referential thinking is also associated with bipolar spectrum disorders [35–40]. Many studies also indicate that negative self-concept is associated with suicidal behavior [41–51]. One specific brain region, much of the medial cortex, is associated with self-referential processing [52, 53]. Some studies have found aberrations in this region to also be associated with suicide [54–57]. Thus, functional abnormalities in medial cortex may be particularly relevant for both self-directed thinking and suicidal behaviors in bipolar spectrum disorders. Studies of this region may be important to completely characterize the neurobiology of these conditions. Furthermore, treatment interventions that target self-referential thinking may have the potential to be efficacious for both decreasing mood symptoms and preventing suicidal behavior. This paper provides an overview of relevant key studies. The goal is to integrate results from diverse lines of investigation and provide a conceptual framework for future studies of self-referential thinking and mindfulness-based interventions in bipolar spectrum disorders.

## 2. Self-Focused Thinking and Mood Disorders

There is evidence that the processing of self-referent information is abnormal in both unipolar and bipolar spectrum illness [35, 39, 58, 59]. For example, anomalous self-schemas (beliefs and ideas about self) form the basis for Beck's classic approach to depression [60]. These cognitive theories suggest that psychological symptoms can occur as a result of dysfunctional schemas [61–63]. Schemas are believed to be lasting cognitive structures held in memory that organize thoughts, emotions and behaviors into stable patterns [61]. It has been hypothesized that dysfunctional self-schemas could lead to negative beliefs about self and thus mood symptoms. Multiple studies have focused on the relationship of negative self-esteem and/or schemas to depression [21–25, 58, 64–73], and the effectiveness of interventions targeting negative schemas through cognitive therapy is well established [4, 6–9]. Finally, self-criticism is associated with depression [74–76]. In regard to bipolar spectrum disorders, many [35–38], but not all [77], investigations indicate that aberrant self-concept/schemas are also a characteristic of these conditions. Altered self-esteem persists during remission [35, 39] including instability of self-concept [36], which may represent a vulnerability to mood episodes. Finally, a recent study found that higher self-esteem corresponded to better prognosis in refractory bipolar disorder [40].

In addition to abnormalities of self-concept, other facets of self-referential thinking are associated with affective disorders. One of these is the amount of time spent thinking about self. Individuals with unipolar illness have increased self-focused thinking [78, 79]. The association between the extent of thinking about self and depressive symptoms is undoubtedly complex, and cause and effect relationships may be difficult to determine. However, excessive self-focus in general is associated with negative affect [80], and high levels of self-focus are thought to contribute to depressive pessimism [81]. Finally, cognitive impairment in depression may be associated with increased self-focus [82]. Therefore increased time spent thinking about self might directly contribute to depressive symptoms, at least in unipolar illness. We are not aware of any studies that have directly addressed this question in bipolar spectrum illness. 

In contrast to the extent of self-referential thinking, there is strong evidence that a particular type of thinking about self contributes to dysphoria in general and unipolar depression. Specifically, analytical self-focused rumination (thinking analytically about self and symptoms) is maladaptive [83]. This cognitive style is associated with overgeneral autobiographical memory [84], global negative self-judgments [85], greater negative future thinking [86], and dysphoria [87, 88]. Furthermore, there is compelling evidence that ruminative self-focus is associated with both the severity and duration of depressive symptoms [89–95] as well as relapse of illness [96]. There is less research investigating the role of rumination in bipolar spectrum illness, but some evidence suggests that rumination is associated with these conditions as well [97, 98]. Importantly, one study [97] found that rumination predicted the number of depressive episodes, which suggests that treatments targeting rumination might be effective for relapse prevention in bipolar spectrum illness.

In summary, considerable evidence indicates that alterations of self-referential thinking are associated with both unipolar and bipolar spectrum illness. One component is negative self-concept, which is likely associated with depression, regardless of diagnosis. Another facet is rumination about self. In particular, analytic self-focused rumination is maladaptive and may represent a treatment target for bipolar spectrum depression. The implications of this will be discussed in the sections that follow.

## 3. Self-Focused Thinking, Rumination, and Suicide-Related Behaviors

As described above, cognitive theories suggest that suicidal ideation, and other psychological symptoms, can occur as a result of dysfunctional self-schemas and thus negative self-focused thinking [61–63]. As with symptoms of depression, there is considerable evidence of an association between self-referential thinking and suicidal behaviors. Numerous studies have reported an association between self-concept and suicide-related behaviors in a variety of populations [41–51]. Most, but not all [99], suggest that low self-esteem and/or negative beliefs about self are risk factors for suicide or suicidal ideation. In contrast, evidence suggests that positive self-appraisals may confer resilience to suicide [100]. Additionally, individuals who attribute negative life events to external, transient, and specific factors are at decreased risk of suicidal ideation as compared to those who explain undesirable outcomes on internal, stable, and global self-characteristics [101]. However, the relationship is complex because there is evidence that self-esteem is decreased by suicide ideation and suicide attempt history [102]. Therefore a dynamic association may exist such that negative self-concept and suicidal behaviors are mutually reinforcing over time whereas positive self-referential thinking inhibits suicidal thinking/behaviors.

In addition to low self-esteem, other aspects of self-referential thinking patterns have been shown to be associated with suicide. For example, suicide has been posited to serve as an escape from awareness of self [103] and research provides support for this hypothesis [104–106]. According to this model, outcomes that fall short of one's standards and expectations for self are interpreted (self-criticism) as personal inadequacies. This attribution causes self-awareness to become painful and generate negative affect. Suicidal thoughts and behaviors can then occur in response to the individual's desires to escape from both self-awareness and the associated unpleasant affect. Thus, a personality trait of higher levels of self-criticism is likely a risk factor for suicide-related behaviors [104, 106].

Automatic self-associations are another important facet of self-referential thinking. Both automatic associations and explicit beliefs reflect aspects of dysfunctional self-schemas likely related to suicidal thinking. Automatic associations reflect simple associations in memory that activate links between self and specific concepts as opposed to the more complex development of explicit beliefs [107]. It has been shown that automatic self-associations of depression and anxiety are significantly related to both suicidal ideation and past suicide attempts [61]. 

Automatic self-associations are clinically relevant because they are associated with both symptom expression [108, 109] and uncontrolled behaviors [108]. Thus, these associations are thought to drive some spontaneous psychopathological behaviors and contribute to the persistence of psychopathological symptoms [61, 110]. The association between dysfunctional automatic self-associations and suicidal ideation [61] therefore suggests that these self-associations may contribute to the onset and maintenance of suicidal ideations as well as the difficulty of controlling these thoughts. Automatic associations of self with death are also associated with suicide [111], which suggests another link between this type of memory processing and suicidal thoughts and behaviors.

Finally as is the case for depressive symptoms, there is compelling evidence that rumination is directly associated with suicidal ideation [112–118]. Furthermore, ruminative thinking may partially mediate relationships between both self-criticism [117] and negative life events [118] with suicidality. Rumination leads to greater negative future thinking [86] which might also contribute to suicidal ideation.

The literature reviewed above provides strong evidence that aspects of self-referential thinking are related to suicidal ideations and behaviors in a variety of populations. Few studies have directly addressed this topic among subjects with bipolar spectrum disorders. However, some evidence indicates that facets of self-focused thinking are relevant to suicide in these conditions. For example, low self-esteem lasting into remission seems related to the expression of suicidality during depressive episodes of bipolar patients [119]. Another study suggests that increased ruminations may mediate the association between anxiety and suicidal ideation/behaviors [120]. Future studies are warranted, as it seems likely that self-referential thinking may be related to the expression of depressive symptoms and suicidality irrespective of diagnosis.

## 4. The Functional Architecture of Self-Focused Thinking in Mood Disorders

Most of the anterior and posterior midline cortex has been characterized as an anatomical and functional unit known collectively as the cortical midline structures (CMS) [52]. The CMS are thought to be important in affective disorders because of anatomical connectivity with the amygdala [121–125] and striatum [126], both of which are important for emotional processing. Further, the CMS has extensive involvement in both self-referential [52, 53] and emotional processing [127, 128], and these regions are components of the default mode network [129, 130].

Studies using a variety of methodologies suggest that functional alterations exist in the CMS in both unipolar [131–145] and bipolar [146–158] spectrum disorders. More importantly, a growing body of evidence directly links the CMS with both self-referential processing and emotional dysregulation associated with depression. A study of healthy controls demonstrated that self-referential processing activates the CMS and that this neural response is associated with negative affectivity [159]. In unipolar illness, studies indicate that abnormal self-referential processing is mediated by neural response in cortical and subcortical midline structures [134, 160]. Abnormal CMS neural response during emotional processing has also been demonstrated in unipolar illness [127, 160]. Furthermore, depressed subjects have increased neural activity during rumination in the CMS, amygdale, and other regions [161]. Finally, depressive symptoms are associated with the degree of CMS activity during a self-negative judgment task [160]. Thus, a growing body of evidence directly links CMS function to self-referential thinking and rumination in unipolar illness, and it appears likely that CMS structures play a key role in mediating the relationship between excessive self-referential thinking and negative affect. 

The potential role of the CMS in emotional dysregulation and self-referential thinking is not as well characterized in bipolar spectrum disorders. However, a number of function imaging studies using emotional activation paradigms suggest a relationship between CMS function and emotional dysregulation in depression [147, 148, 155], mania [156–158], as well as euthymia [162]. Most investigations have studied bipolar I disorder; however, our group recently reported evidence of CMS-mediated emotional dysregulation in bipolar II disorder [153]. Finally, there is also some evidence of changes in CMS activation associated with treatment response [152, 163–165]. To our knowledge, aberrant self-referential processing in bipolar disorder has not yet been investigated using functional neuroimaging methods.

In summary, compelling functional imaging evidence indicates that CMS dysfunction in unipolar illness is associated with self-referential thinking and emotional regulation in unipolar illness. Further, the CMS likely mediates interactions between self-focused cognitions and emotional symptom expression. In bipolar disorders, considerable evidence indicates that CMS function is related to emotional dysregulation. Neuroimaging studies of self-referential processing in bipolar spectrum conditions are warranted.

## 5. The Role of the Striatum and CMS in Suicide-Related Thoughts and Behaviors

A large literature documents the role of striatal and associated corticobasal ganglia circuitry dysfunction in affective disorders, see [166, 167] for recent reviews. Recent studies also implicate striatal dysfunction in the neurobiology of suicide-related thoughts and behaviors in both bipolar [168] and unipolar spectrum disorders [169–171] as well as among those with alcohol dependence [172]. In regard to the CMS, there is structural, functional imaging [54] and molecular [55–57] evidence that dysfunction in this region may also be related to suicide.

The striatum and CMS have extensive anatomical [126, 173] and functional [174] connectivity. Importantly, striatal-CMS connectivity has been shown to be disrupted in bipolar II depression [168]. Finally, aberrant functional connectivity of the striatum with the posterior medial cortex has been shown to correlate with depression severity [168].

Taken together, these studies suggest that both CMS and striatal dysfunction may be relevant for suicide. Future investigations of the potential role of these regions in suicide-related behaviors are warranted. In particular, additional investigations of functional connectivity between the two regions may provide important insights.

## 6. Implications for Studies of Self-Referential Processing and Suicide in Bipolar Spectrum Depression

A primary aim of this manuscript is to integrate results of diverse key studies in order to provide a conceptual framework for future investigations of bipolar depression. Conclusions from the investigations reviewed herein are summarized in Table 1.

It is now well established that aspects of aberrant self-referential thinking contribute to the symptoms of unipolar depression. The strongest evidence is for analytical self-focused rumination. However, the amount of time spent thinking about the self may also be relevant. Studies clearly demonstrate that CMS play primary role in self-referential thinking. Additionally, there is strong evidence the CMS dysfunction plays a direct role in emotional dysregulation in unipolar depression and likely mediates the relationship between self-focused thinking and emotional dysregulation in this disorder. Finally, abnormalities of both the striatum and CMS may contribute to suicide risk in affective illness and the functional connectivity between the new regions may be of particular relevance.

In regard to bipolar disorder, there is relatively strong evidence of aberrations of both self-referential thinking and CMS function. Further, CMS dysfunction appears to be associated with deficits of emotional regulation. It is unclear if self-focused thinking and emotion dysregulation are linked by the CMS, as is the case for unipolar depression. However, this possibility clearly warrants investigation. Studies of the potential roles of both the striatum and CMS in suicide are indicated as well.

Future studies, as suggested above, may enhance our understanding of the neurobiology of bipolar depression. More importantly, it is possible that a subregion of the CMS might mediate relationships between self-referential thinking, emotional dysregulation, and suicidality. Such a finding would likely be very relevant for the development of biomarkers as well as treatment interventions.

## 7. Implications for Bipolar Depression Treatment Studies Using Mindfulness Interventions

The studies reviewed herein have implications for studying neurobiological mechanisms in bipolar depression as described above. More importantly, these investigations support studies of mindfulness-based treatment interventions that specifically focus on altered self-referential processing.

There is not yet complete consensus as to how the concept of mindfulness should be properly operationalized in Western psychology, and such lack of consensus is reflected into the multiplicity of definitions of mindfulness employed by different authors concerned with mindfulness-based interventions, for example [175]. However, mindfulness is usually described as a practice through which one learns to focus attention on moment-by-moment experience with an attitude of curiosity, openness, and acceptance. Practicing mindfulness is simply experiencing the present moment, without trying to change anything [20, 176]. During mindfulness, awareness is focused on external sensory inputs, such as auditory, olfactory, and visual stimuli, as well as internal sensations, such as proprioception and pain. Furthermore, attention is specifically focused on awareness of the internal workings of the mind [20].

Although an increasing number of mindfulness-based interventions are currently employed for a large variety of psychiatric disorders, two such interventions, mindfulness-based stress reduction (MBSR) and mindfulness-based cognitive therapy (MBCT), have proven to be particularly fruitful approaches for many of these conditions. The concept of mindfulness originated in Buddhist spiritual practices [177]; however, both MBSR and MBCT are secular, clinically based methods utilizing manuals and standardized techniques. MBSR was developed by Dr. Kabat-Zinn at the University of Massachusetts Medical Center [20]. MBSR includes education about stress as well as training on coping strategies and assertiveness in addition to mindfulness. The mindfulness component includes sitting meditation, a body scan (focusing on bodily sensations), and Hatha Yoga. MBSR involves the cultivation of a number of attitudes, including becoming an impartial witness to one's own experience and acceptance of things as they actually are in the present moment [20]. MBCT was developed by Segal et al. [178]. MBCT is based upon MBSR and combines the principles of cognitive therapy with those of mindfulness to prevent relapse of depression. MBCT, like MBSR, utilizes secular mindfulness techniques including seated meditation. The program specifically teaches recognition of deteriorating mood with the aim of disengaging from self-perpetuating patterns of ruminative, negative thought that contribute to relapse [178].

A very large literature supports the effectiveness of MBSR on several psychological outcomes. Examples include studies of social anxiety disorder [179], generalized anxiety disorder [180], insomnia [181], psychological functioning among individuals with a large variety of medical disorders [182–186] including fibromyalgia [187, 188], cancer [182, 186, 189–192], gay men living with HIV [193], and pain [183, 187, 194–197] as well as healthy individuals [198]. Evidence indicates that MBCT is beneficial for unipolar depression relapse prevention [199–204], generalized anxiety disorder [180], panic disorder [205], hypochondriasis [206], and social phobia [207]. The strongest evidence is for relapse prevention in unipolar illness. A recent meta-analysis [208] concluded that MBCT was significantly better than usual care for reducing relapses in those with three or more prior episodes. Additional conclusions were that effectiveness for relapse prevention was similar to antidepressants at one year and that augmentation with MBCT could be useful for reducing residual depressive symptoms. A subsequent study [204] provided compelling evidence confirming that for depressed patients, MBCT offers protection against relapse equal to that of maintenance antidepressant pharmacotherapy. Further, a recent review and meta-analysis [15] specifically addressed the effectiveness of MBSR and MBCT (and similar interventions) for reducing symptoms of anxiety and depression. The authors concluded that mindfulness-based therapy improves symptoms of anxiety and depression across a wide range of severity and even when these symptoms are associated with other disorders [15]. This study supports the use of these interventions for acute treatment as well as relapse prevention. Finally, there is evidence of effectiveness of MBCT for depression among both symptomatic and partially symptomatic patients [209].

These studies suggest that both MBSR and MBCT have broad-spectrum antidepressant and antianxiety effectiveness. Furthermore, preliminary evidence suggests that MBCT is feasible for use in bipolar disorder and may decrease symptoms of anxiety and depression [17–19]. Of note, one of these investigations [17] specifically studied individuals who had previously experienced serious suicidal ideation. Finally, evidence is accumulating in support of the use of mindfulness interventions for suicide prevention [210–213], and additional research is ongoing [214].

The studies citied above indicate that additional trials of mindfulness interventions for bipolar spectrum disorders are warranted. In particular, studies of acute and maintenance treatment of depressive and anxiety symptoms as well as suicide prevention are warranted.

The possibility that mindfulness interventions may help prevent suicide is particularly intriguing. In bipolar spectrum disorders, severity of affective episodes [215] comorbid panic [216], and psychosocial stress [217] are suicide risk factors. Thus, mindfulness interventions might decrease suicide risk through more than one mechanism.

## 8. Neuroimaging of Mindfulness: Implications for Studies of Bipolar Depression

Neuroimaging studies of mindfulness add to the evidence supporting trials of mindfulness interventions for bipolar depression. Furthermore, these studies suggest specific investigations that could be aimed at exploring the neural mechanisms underlying any observed benefits.

As described above, the CMS play a key role in both self-referential and emotional processing [52, 53, 127, 128]. Further, evidence indicates that CMS structures play a direct role in mediating the relationship between self-referential thinking and negative affect in mood disorders [127, 134, 147, 148, 153, 155–162]. A number of structural and functional neuroimaging as well as electroencephalogram (EEG) studies have provided information about the neural processes underlying mindfulness practices. Some key findings are reviewed herein, for a more detailed recent review, see [218]. 

Zen meditation is a mindfulness practice that has been studied using neuroimaging methods. Zen is a traditional Buddhist approach to mindfulness [219, 220]. Zen primarily involves the practice of developing mindfulness by way of seated meditation [219, 220]. During meditation periods, known as Zazen, practitioners sit silently without moving on either a cushion or in a chair [220]. It is important to note that while Zen meditation is a Buddhist practice, it is frequently practiced as a secular means to achieve mindfulness [221].

A number of investigations indicate that Zen meditation induces changes in brain function measurable by both functional MRI (fMRI) and EEG [222–228]. One fMRI study [228] of regular Zen practitioners and matched control subjects found that Zen practitioners displayed a reduced duration of the neural response linked to conceptual processing in regions of the default network, including a portion of the CMS. The authors concluded that that meditative training fosters the ability to control the automatic cascade of semantic associations triggered by a stimulus or, in other words, voluntarily regulate the flow of spontaneous mentation. This study suggests that the practice of Zen meditation may facilitate the ability to regulate self-referential thinking by consciously modulating the function of the CMS and other brain regions.

In addition to functional changes, evidence also indicates that extended Zen practice is associated with changes in brain morphology. In one study [229], structural MRI scans were performed and pain tolerance was assessed in meditators and controls. Meditators had significantly lower pain sensitivity than controls. Furthermore, meditators were found to have thicker cortex in a CMS region (dorsal anterior cingulate) and other areas and more years of meditation experience was associated with thicker gray matter in the anterior cingulate. This study provides compelling evidence that a mindfulness practice can impact CMS structure in addition to effects on function as demonstrated by other studies [222–228]. Another study [230] examined how the regular practice of Zen meditation might affect the normal age-related decline of cerebral gray matter volume and attentional performance observed in healthy individuals. Structural MRI and a computerized sustained attention task were used to study regular practitioners of Zen meditation and controls. Control subjects displayed the expected negative correlation of both gray matter volume and attentional performance with age. However, in contrast, meditators did not show a significant correlation of either measure with age. The effect of meditation on gray matter volume was most prominent in the putamen. The authors conclude that these findings suggest that the regular practice of meditation may have neuroprotective effects and reduce the cognitive decline associated with normal aging.

Several studies of mindfulness and other meditation practices also indicate these methods impact CMS function [231–235]. For example, Vipassana is a mindfulness technique that has similarities to Zen meditation, but has a particular focus on introspection, contemplation, and the development of insight [236]. An fMRI study [235] of this practice also revealed differences in CMS activation among meditators as compared to controls.

In addition to the investigations described above, MBSR has also been studied using functional and structural neuroimaging. One study [237] found that prior to MBSR training, experiential focus (mindfulness) was associated with decreased CMS activation as compared to narrative focus. After MBSR training, experiential focus resulted in marked and pervasive reductions of CMS activation along with increased engagement of other regions. Further analyses demonstrated uncoupling of right insula and CMS connectivity in response to MBSR. This study provides compelling direct evidence that neural process underlying mindfulness involves both CMS activation and connectivity with other regions. Another study [238] recently demonstrated that this practice resulted in altered connectivity between anterior CMS regions and sensory cortex, which may indicate greater reflective awareness for sensory experience as a result of MBSR. In regard to structural imaging, recent studies [239, 240] indicate that MBSR, like Zen, also alters brain morphology. One study [239] found changes in gray matter concentration in the CMS as well as left hippocampus, the temporoparietal junction and the cerebellum. Thus in addition to impacting the CMS, mindfulness practices may decrease emotional reactivity by modulating other mechanisms as well. At least two other studies also support this conclusion. A recent study [179] reported decreased amygdala activation as a result of patients receiving MBSR for social anxiety disorder. Another study indicated that reductions in perceived stress correlated positively with decreases in right basolateral amygdala gray matter density in response to MBSR [240].

As described above, compelling evidence now exists demonstrating that mindfulness practices impact both the structure and function of the CMS as well as other regions. Furthermore, sadness is associated with CMS activation and mindfulness training changes the neural response associated with this emotion [241]. Taken together, the studies reviewed herein suggest a hypothesis of the neural mechanisms underlying any beneficial effects mindfulness practices may provide for bipolar depression. Specifically, these interventions likely exert benefit, at least in part, by modulating CMS functions associated with both self-referential thinking and emotional regulation. Thus, studies of mindfulness practices for bipolar disorder may benefit from a functional imaging component aimed at exploring CMS activation and connectivity. Such studies should also evaluate striatal function because meditation impacts this region [230, 232] as well as the fact that dysfunction of this area is associated with both affective illness [166, 167] and suicide [54–57, 168–172].

## 9. Conclusions

Bipolar disorder is a disabling [242–244] and difficult to treat [245–248] condition. In particular, bipolar depression is a source of considerable suffering [249, 250] and is often associated with anxiety [251, 252] and increased suicidal risk [249, 250, 253, 254]. Current treatments are often only partially effective [2], and relapse is a common occurrence [2, 3]. Adjunctive psychotherapy approaches are needed that are effective for depressive and anxiety symptoms as well as suicide prevention.

Converging lines of clinical evidence reviewed herein indicate that mindfulness-based interventions, such as MBSR and MBCT, hold great promise as psychotherapeutic interventions for bipolar depression. From the clinical perspective, there is compelling evidence that MBSR and MBCT have broad-spectrum effectiveness for anxiety and depressive symptoms [15, 179–209]. Additionally, evidence now specifically indicates that MBCT is feasible for use in bipolar disorder and decreases symptoms of both anxiety and depression [17–19]. Finally, evidence is accumulating in support of the use of mindfulness interventions for suicide prevention [210–213]. Thus, investigations of mindfulness-based interventions for bipolar spectrum disorders are clearly warranted as these approaches have the potential to decrease symptoms of depression and anxiety as well as suicide risk.

In addition to the clinical evidence, psychological and neurobiological studies come together suggesting a hypothetical mechanism of action for mindfulness practices in the treatment of bipolar disorders. Specifically, the processing of self-referent information is abnormal in affective disorders [35, 39, 58, 59]. The CMS play a key role in both self-referential [52, 53] and emotional processing [127, 128], and a growing body of evidence indicates that CMS structures play a direct role in mediating the relationship between self-referential thinking and negative affect in mood disorders [127, 134, 147, 148, 153, 155–162]. Mindfulness interventions target aberrant self-referential thinking [20], and neuroimaging studies indicate that mindfulness practices impact both structure and function of CMS [228, 229, 231–235, 237–239]. Thus, these interventions likely exert benefit, at least in part, by modulating CMS functions associated with both self-referential thinking and emotional regulation. Studies of mindfulness practices for bipolar disorder should include a functional imaging component aimed at exploring CMS activation and connectivity. These studies should also evaluate striatal function because meditation impacts this region [230, 232] as well as the fact that dysfunction of this area is associated with both affective illness [166, 167] and suicide [54–57, 168–172]. Such investigation could provide important insights into how mental training can affect the cognitive and emotional dysfunctions typically observed in patients with affective disorders.

## Figures and Tables

**Table 1:** Conclusions from studies reviewed.

(i) Abnormalities of self-referential processing are likely a general characteristic of mood disorders	
(ii) Increased self-focused thinking may contribute to depressive symptoms	
(iii) Analytical self-focused rumination clearly contributes to the manifestation of depression in unipolar illness and possibly in bipolar spectrum depression	
(iv) Aspects of aberrant self-focused thinking (particularly rumination) likely contribute to suicide-related behaviors and thoughts in unipolar depression	
(v) The cortical midline structures (CMS) play a key role in both self-referential thinking and emotional regulation	
(vi) Functional abnormalities of the CMS are likely a characteristic of mood disorders in general	
(vii) In unipolar illness, CMS function plays a role in dysregulation of both self-referential thinking and emotion and likely mediates the relationship between the two	
(viii) Dysfunction of both the striatum and CMS are likely involved in the neurobiology of suicide-related thoughts and behaviors	
(ix) Abnormal functional connectivity between the striatum and CMS may be relevant for both the expression of both depressive symptoms and suicide	
